# When narratives speak louder than numbers: the effects of narrative persuasion across the stages of behavioural change to reduce air pollution

**DOI:** 10.3389/fpsyg.2023.1072187

**Published:** 2023-04-26

**Authors:** Lucia Bosone, Marie Chevrier, Frédéric Martinez

**Affiliations:** ^1^Université Gustave Eiffel, Université Paris Cité, LaPEA, Versailles, France; ^2^Equipe Mobilité Durable, Individu, Société, Université Gustave Eiffel, Lyon, France

**Keywords:** air pollution, psychological distance, narrative persuasion, stages of behavioural change, efficacy appraisal

## Abstract

Is narrative persuasion effective when promoting new behaviours in favour of the environment? Does this effectiveness vary depending on whether individuals are already thinking about changing? This paper has two main objectives: (1) to explore how individuals at different stages of the behavioural change process perceive air pollution, focussing on the perceived psychological distance of its environmental risks (Study 1); and (2) to test whether the effects of presenting the risks of air pollution in a narrative vs. statistical format on pro-environmental intentions vary depending on the individuals’ stage of behavioural change (Study 2). Study 1 (*N* = 263) is based on a survey measuring individuals’ perceived psychological distance of the environmental risks of air pollution, and the perceived effectiveness of different pro-environmental behaviours. Results suggest that perceived distance and perceived effectiveness vary across different stages of behavioural change. Study 2 (*N* = 258) presents a 2(Format: narrative vs. statistical) × 3(Stages of change) protocol, testing the effectiveness of a narrative format depending on individuals’ stage of behavioural change. Results suggest that proximising a threat through a narrative format of communication is more effective especially for individuals in the pre-action stage of change. We also present a moderated mediation model explaining the influence of the interaction between the message format and the stage of behavioural change on behavioural intentions and on efficacy appraisal via narrative engagement. Findings are discussed with regards to the stage model and narrative persuasion.

## 1. Introduction

The air pollution levels that we observe today are the consequence of increased global industrialisation and urbanisation. The large-scale industries that introduce the majority of pollutants into the air are transportation, industrial machinery, energy production and modern agriculture (World Health Organization, 2019). There is also some personal accountability for individuals who use personal motorised vehicles, household energy, waste disposal and the excessive consumption of luxury goods. These personal behaviours are increasingly contributing to air pollution as the scale of this behaviour increases in both developed and developing countries (Carlsten et al., 2020), which has negative effects for the climate, ecosystems, and citizens’ health. Air pollution is defined by the World Health Organisation as “the contamination of the indoor or outdoor environment by any chemical, physical or biological agent that modifies the natural characteristics of the atmosphere.” Six major pollutants have been identified: ground-level ozone, carbon monoxide, particle pollution, sulphur oxides, nitrogen oxides, and lead. Although the main effects of these pollutants concern the health of individuals as well as all living organisms and the ecosystems in general (through the acidification of water and soil; Manisalidis et al., 2020), air pollution is also strongly linked to climate change. Some minor air pollutants, such as methane and black carbon, are powerful short-lived climate pollutants (SLCPs), which have notable global warming potential even if they persist in the atmosphere for short lifetimes. Moreover, the main sources of air pollution, such as the use of fossil fuels (e.g., in the field of transportation), are also sources of high CO_2_ emissions, which are strictly linked to increasing temperature and extreme weather events (NASA Vital Signs; Crowley and Berner, 2001; Amirkhani et al., 2022). The WHO thus explains how “reducing ambient and household air pollution can also reduce emissions of carbon dioxide (CO_2_) and short-lived climate pollutants, therefore contributing to the near- and long-term mitigation of climate change.”

Although levels of air pollution density have peaked and started to decline following air quality initiatives in developed countries, many developing countries are heading toward higher levels, adding strain to already dangerous climate conditions (Ritchie and Roser, 2019). There has been some progress, with the proportion of the European population exposed to ground-level ozone reducing from 54% in 2007, to 7% in 2014; however, following increased temperatures in 2018, an estimated 30% of the population was exposed to levels above targets (European Environment Agency, 2018). In 2018 15% of urban citizens were also exposed to values of Particulate Matter above targets and 4% to limit-exceeding levels of NO_2_ (European Environment Agency, 2018). Therefore, despite progress to reduce emissions in European countries, air quality still needs to be effectively and consistently reduced to safe levels.

Public perceptions of air pollution and the associated risks are thus crucial for meeting the emissions targets of countries worldwide, particularly those of Western countries where targets are challengingly low. It is therefore important that we understand how people perceive air pollution, in order to identify effective communication strategies to use in information and education programmes and campaigns to motivate citizens to change their behaviour in order to improve air quality.

The present research has two main objectives. The first one (pursued in Study 1) is to explore the public perception of the environmental risks of air pollution, and whether this might drive individuals through the different stages of behavioural change. The second objective (pursued in Study 2) is to test whether presenting the risks of air pollution in a narrative format is more effective than a statistical format when promoting pro-environmental behaviours to improve air quality, depending on individuals’ stage of behavioural change.

### 1.1. Social perception of air pollution through the stages of behavioural change

The majority of the studies exploring the public perception of air pollution focus on individuals’ subjective evaluations of the pollution levels where they live, and the risks that this can have with regards to their health (Bickerstaff and Walker, 1999, 2001; Bush et al., 2001a,b; Howel et al., 2003). Although there are no studies specifically concerning individuals’ perception of the environmental risks of air pollution, it has been demonstrated that individuals generally tend to represent environmental risks such as climate change (Brulle et al., 2012; Spence et al., 2012; Kohut and Pew Research Center, 2013; Leiserowitz et al., 2013; Stoknes, 2014) and biodiversity loss (Bosone and Bertoldo, 2022) as distant threats. These phenomena are indeed perceived as psychologically distant (Trope and Liberman, 2010; Spence et al., 2012), something happening in a distant future (a sub-dimension defined as temporal distance), to other people (social distance), in faraway geographical areas (geographical distance) and with a relative degree of uncertainty (uncertainty).

Distantiating risks on these dimensions is a process that individuals adopt to protect themselves as this allows them, on the one hand, to feel more secure and, on the other hand, to avoid changing their behaviours which are contributing to the issue they are distantiating from. However, such a process is problematic as it entails a lesser concern for important issues (Brügger et al., 2015) and as a consequence, a weaker engagement in behavioural change in favour of the environment (Creutzig et al., 2018). It is thus possible to suppose that different degrees of perceived psychological distance would characterise individuals’ different positions with regards to behavioural change, also definable as different stages of the process of behavioural change.

Indeed, behavioural change should not be considered as an on-off switch, but rather as a dynamic process based on several stages of change, where individuals can advance but also retrocede. The process of change has been described by the *trans*-theoretical model (TTM; Prochaska and DiClemente, 1994), which considered behavioural change as a transition through five stages. Individuals in the first stage of “pre-contemplation” do not intend to take action and change their behaviour in the foreseeable future. Individuals in the second “contemplation” stage become aware of the necessity for a change in their behaviour. Individuals in the third “preparation” stage form the intention to change their behaviour in the foreseeable future, and when they actually initiate the new behaviour they pass in the fourth “action” stage. The fifth and final “maintenance” stage includes individuals who are already engaged in the new behaviour and work to prevent relapse. Past research in the field of health-promotion has demonstrated how different strategies are more or less effective in pushing individuals through different phases; for instance, while risk vulnerability and severity seems to increase intention to engage in the targetted behaviour for individuals in the pre-contemplation and contemplation stages, perceived efficacy of the targetted behaviour and self-efficacy increase behavioural intentions for individuals in the action stage (Horowitz, 2003; Martin et al., 2007; Bočkarjova et al., 2009; Lopes et al., 2016; Mohebbi et al., 2021). It is possible to suppose that this is due to the fact that individuals perceive risks and behavioural effectiveness differently in the different stages. Several studies in the field of health-promotion have for instance demonstrated that the different stages of the *trans*-theoretical model are characterised by different combinations of the threat- and coping-appraisal processes described by the Protection Motivation Theory (e.g., Maddux and Rogers, 1983). Threat-appraisal refers to individuals’ perception of the vulnerability and severity of the risk they are facing, whereas coping-appraisal refers to their perception of the effectiveness of the possible behavioural alternatives available to cope with such a threat and of their own ability to adopt them (i.e., self-efficacy). In the meta-analysis carried out by Marshall and Biddle (2001) on the stages of behavioural change with regards to dietary issues, individuals in a stage of pre-contemplation perceived substantially lower scores for both threat appraisal (perceived vulnerability and severity) and coping-appraisal. While a linear increase of coping-appraisal (specifically, self-efficacy) was observed between the preparation, action and maintenance stages, threat appraisal constructs showed a non-linear association with stage progression. More precisely, individuals in pre-contemplation had significantly lower levels of consciousness than did individuals in all other stages, which increased slightly for individuals in the contemplation stage, and did not differ between the action and maintenance stages. The most recent research on the TTM commonly consider risk perception and awareness as levers in the field of health promotion to increase the intention of individuals’ in the pre-contemplation stage to adopt a new behaviour (e.g., de Freitas et al., 2020; de Carvalho et al., 2021), without measuring the actual risk perception or efficacy perception of individuals in the different stages. More importantly, while the stage model of behavioural change has been validated and tested in the field of health-promotion, it is still relatively new in the field of pro-environmental behaviour (Bamberg, 2013; Andersson, 2020). We argue that individuals in different behavioural change stages with regards to pro-environmental behaviours will report different degrees of the perception of the risk of the environmental consequences of air pollution. More precisely, we expect individuals in the pre-action stages (pre-contemplation and contemplation) to perceive the environmental consequences of air pollution to be at a higher psychological distance, than individuals in the following stages. Similarly, we expect them to also report lower perceived effectiveness of possible behaviours to cope with air pollution than individuals in the following stages. The goal of our first study is thus to explore whether individuals’ perceived psychological distance of the environmental risks of air pollution, as well as their perception of the effectiveness of possible pro-environmental behaviours to reduce air pollution, varies according to the individuals’ stage of behavioural change.

The fact that perceived risk and perceived effectiveness are key elements pushing individuals through the phases of behavioural change is particularly important for its practical implication, as it would confirm that one of the levers that educational and communication programmes could use to push individuals to advance in the process of behavioural change is “proximising” the risks of air pollution while at the same time increasing individuals’ perception of the effectiveness of behavioural changes improving air quality.

### 1.2. “Proximising” the risks by narrative persuasion

A few studies have tried to use different communication techniques to “proximise” the risks of climate change, by describing it as an issue that is geographically close to the targetted individuals and as particularly concerning the social group to which they belong (Shwom et al., 2008; Spence and Pidgeon, 2010; Schuldt et al., 2018; Loy and Spence, 2020). However, these studies showed that such a technique did not have a significant effect on behavioural intentions, or behavioural change (Brügger et al., 2015, 2016).

We argue that this depends on the strategy that is used to proximise psychological distance. Indeed, within the studies that did not find a significant influence of proximising psychological distance in persuasive messages, the environmental risks are presented through scientific data on climate change, thus adopting a numerical approach of presentation of risks. Indeed, past research has demonstrated that presenting information in a narrative format is more effective than presenting information in a statistical format (Rothman and Kiviniemi, 1999; de Wit et al., 2008; Miller-Day and Hecht, 2013), especially in areas such as health, advertising, and education (Hornikx, 2005; De Graaf et al., 2016).

Narrative evidence has been studied with regards to its effects on support for controversial political policies (Igartua, 2010), health behaviours (Hinyard and Kreuter, 2007; Shen, 2015), and recruitment into extremist groups (Casebeer and Russell, 2005; Braddock, 2015). A few studies, however, reported non-significant effects of narratives on intentions and behaviours (e.g., Cheney et al., 2006; Prati et al., 2012), or even opposite effects to those expected (e.g., Gesser-Edelsburg and Endevelt, 2011). In two recent meta-analyses, a positive association between narrative evidence and intentions and behaviours emerged both in the specific field of health communication (Shen, 2015), and more in general in persuasive communication (Braddock and Dillard, 2016). Although the use of narratives in environmental education interventions is rather frequent (such as the use of movies and documentaries in what is defined as entertainment education; see Bahk, 2010; Topp et al., 2019), little research has tested the persuasive effects of narrative evidence compared to other types of evidence format. In a recent experimental study by Nakano and Hondo (2023), where participants received either narrative or logical information about climate change and completed several measures related to behavioural intentions, interest, and emotions, results indicated that a narrative format triggers stronger negative emotions and behavioural intentions, especially for individuals who reported having a low interest in the issue. Based on this literature review, we argue that narrative evidence is more effective than statistical evidence in reducing the perceived psychological distance of the environmental risks, and in increasing pro-environmental behavioural intentions, but only for individuals in the pre-action stage of behavioural change.

Hypothesis 1 (HP1; tested in Study 2) is thus the following: presenting the environmental risks of air pollution in a narrative format will increase individuals’ intentions to engage in pro-environmental behaviours as compared to presenting them in a statistical format, but only for individuals in the first stage of behavioural change (pre-action). Indeed, since in this stage individuals are less interested and worried about the targetted issue, a narrative message will be more effective than a statistical one. On the contrary, we expect individuals in the action and post-actrion stages to be already concerned by the environmental risks of air pollution, and thus to be less sensitive to the influence of the information format.

But why will narrative evidence be more persuasive than statistics? A narrative message engages individuals through the message being reported and transports them into the story (Busselle and Bilandzic, 2008; Sestir and Green, 2010), creating what can be defined as “narrative engagement” (De Graaf et al., 2009). Once immersed in a narrative, it is argued that mental-processes are focussed upon the narrative being presented, resulting in less counter-argument and attention to the message’s potential faults (Slater and Rouner, 2002). By forming vivid mental simulations of events, narrative information can enable the formation of an alignment with the protagonist persuading greater levels of acceptance of the message’s intended response (Green and Clark, 2013), and thus mediating the effectiveness of message adoption and behavioural change (Lee et al., 2011). We argue that the effectiveness of presenting the risks of air pollution in a narrative format will partly depend on the influence such a format has on individuals’ efficacy appraisal, including self-efficacy and response-efficacy (Bosone et al., 2015). Self-efficacy refers to a person’s belief about their ability to engage in a behaviour and is often associated with response efficacy, which is the perceived effectiveness of one’s behavioural change (Witte and Allen, 2000). As Bandura (1982) posits, a greater awareness and knowledge of risks is an important precursor to changing behaviour yet it is not sufficient. There need to be means, resources and social support to mitigate unwanted behaviours. He determines that successful changes require a strong belief in one’s ability to use self-regulatory skills effectively. The use of narrative information is predicted to improve the self-efficacy experienced by the subject, as Bandura (1982) determines that vicarious experiences, such as those from an engaging narrative, are a potential source of self-efficacy. It is also possible to argue that narrative messages will increase perceived response-efficacy, as individuals transported into a narrative engage in mental simulations of how they would behave in a situation like the one described (Escalas, 2004; Nielsen et al., 2018).

Based on past research on the effects of narrative evidence on individuals’ engagement, we expect the persuasiveness of a narrative vs. statistical format, moderated by the stages of behavioural change, to be mediated by individuals’ engagement with the message, influencing efficacy appraisal as well as perceived psychological distance and behavioural intentions. Hypothesis 2 (HP2; tested in Study 2) is thus the following: the effects of narrative vs. statistical format on behavioural intentions is mediated by individuals’ engagement with the message, and this mediation is moderated by the stages of behavioural change.

### 1.3. Objectives

The present research has two main objectives. The first one is to explore the public perception of the environmental risks of air pollution, and whether this might push individuals through the different stages of behavioural change.

To this purpose, by means of a survey, Study 1 explored individuals’ perceived psychological distance of the environmental risks of air pollution, as well as their perception of the effectiveness of several behaviours in improving air quality, with particular attention to the possible differences according to the participants’ stage in the process of behavioural change.

The second objective is to test whether presenting the risks of air pollution in a narrative format is more effective than a statistical format in boosting individuals’ intentions to engage in pro-environmental behaviours to improve air quality, depending on individuals’ stage of behavioural change. To this purpose, Study 2 tested whether the effectiveness of a narrative message depends on individuals’ stage of behavioural change.

The data that supports the findings of the two studies is available from the corresponding author upon reasonable request. All data was collected and analysed according to the latest General Regulation on Data Protection.

## 2. Study 1

### 2.1. Method

#### 2.1.1. Participants

A total of 263 people took part in the study (58.3% women and 41.3% men). Participants ranged in age from 18 to 71 years (*M* = 44.1; *SD* = 12.5), and 93.1% were licenced drivers. Participants were recruited online, by publishing a post on several social network groups. Data was collected between March and April 2021, participants answered the questionnaire on Qualtrics. Participation was voluntary and free, all participants gave their consent to the analysis of their answers.

#### 2.1.2. Materials and procedure

The measures were presented in the order in which they are described below (the questionnaire is reported in the Supplementary material). After the completion of the questionnaire, participants were thanked and fully debriefed.

##### 2.1.2.1. Psychological distance

Individuals’ perception of the psychological distance of the environmental risks of air pollution was measured by adapting the scale of climate change psychological distance [ Spence et al., 2012; α (*N* = 8) = 0.78], asking individuals to rate their agreement with 8 items, measuring the geographical (e.g., “The environmental risks of air pollution are more likely to affect far away countries”), temporal (e.g., “are an important issue right now”), social (e.g., “The environmental problems caused by air pollution will certainly affect me and my family”) and uncertainty (e.g., “I am sure that air pollution has negative environmental consequences”) sub-dimensions of psychological distance, on a seven-point scale from *1- Strongly disagree* to *7- Strongly agree*.

##### 2.1.2.2. Behavioural effectiveness

Participants were asked to rate, on a seven-point scale from *1-Not at all effective* to 7-*Completely effective*, the extent to which they believed a list of twelve pro-environmental behaviours to be effective in improving air quality [α (*N* = 12) = 0.77].

##### 2.1.2.3. Socio-demographic details and behavioural change stage

In the final section of the questionnaire, individuals were asked to indicate their gender, their age, and whether they had a driving licence or not. Moreover, following the method used by Godin et al. (2004) and by Andersson (2020), we measured the behavioural change stage of individuals by asking them to choose what they thought would happen in the 6 months following the survey, among the following items:

• “I do not intend to change my lifestyle in order to help reduce air pollution within the next 6 months” (pre-contemplation).
• “I can imagine myself changing certain habits in order to help reduce air pollution within the next 6 months” (contemplation).
• “I have started trying to change certain parts of my lifestyle in order to help reduce air pollution over the last 6 months” (preparation).
• “During the past 6 months, I have changed my lifestyle in order to help reduce air pollution” (action).
• “For the past 6 months, I have maintained a lifestyle that helps reduce air pollution” (maintenance).

Following their answers, 7.6% of participants were classed in the pre-contemplation stage, 6.5% in the contemplation stage, 28.5% in the preparation stage, 20.9% in the action stage, and 36.5% in the maintenance stage.

### 2.2. Results

To analyse the influence of the stages of behavioural change and considering the uneven distribution of the sample across stages, we decided to consider individuals in the pre-contemplation and in the contemplation stage together, as in a “pre-action” stage, as individuals in both stages are ambivalent about their current behaviour (following the same procedure as in Forward, 2014 and in Andersson, 2020). Participants were thus divided into four groups according to their behavioural change stage: pre-action (*N* = 37), preparation/intention (*N* = 75), action (*N* = 55), and maintenance (*N* = 96). As the sample sizes were still not equally distributed, a Levene’s test was conducted to determine whether the data met the homogeneity of variance assumption. The test infirmed the null hypothesis (*p* < 0.005) that all the stages have similar population variances; consequently, Welch’s ANOVAs were carried out to test the influence of the stages on the continuous dimensions of psychological distance and perceived effectiveness of behaviours.

#### 2.2.1. Perceived psychological distance according to behavioural change stages

The Welch’s ANOVA showed that the general psychological distance of the environmental risks of air pollution varied significantly depending on the stage of behavioural change, *F*_(3, 105)_ = 5.40, *p* = 0.002. More precisely, Games-Howell *Post hoc* test indicated that individuals in the stage of pre-action (*M* = 5.75; *SD* = 1.07) perceived environmental risks of air pollution as less close than individuals in stages of maintenance (*M* = 6.42; *SD* = 0.59, *p* = 0.004).

#### 2.2.2. Perceived behavioural effectiveness according to behavioural change stages

Welch’s ANOVA showed that the perceived effectiveness of actions varied with participants’ stage of change [*F*_(3, 107)_ = 10.74, *p* < 0.001].

Indeed, perceived effectiveness were lower for individuals in the stage of pre-action (*M* = 4.67; *SD* = 1.12) than individuals in the stage of preparation (*M* = 5.50; *SD* = 0.70, *p* < 0.001), action (*M* = 5.73; *SD* = 0.80, *p* < 0.001) and maintenance (*M* = 5.73; *SD* = 0.65, *p* < 0.001) (see Table 1).

**TABLE 1:** Means, standard deviations and Games-Howell test (Study 1).

Measure	Pre-action (a) *N* = 37	Preparation (b) *N* = 75	Action (c) *N* = 55	Maintenance (d) *N* = 96
	*M* (*SD*)	*M* (*SD*)	*M* (*SD*)	*M* (*SD*)
Psychological distance of the risks of air pollution	5.75^a^** (1.07)	6.16 (0.81)	6.27 (0.80)	6.42^b^** (0.59)
Perceived efficacy of behaviours for improving air quality	4.67^abc^*** (1.12)	5.50^d^*** (0.70)	5.73^d^*** (0.80)	5.73^d^*** (0.65)

Mean score with different letters are Games-Howell pairwise comparisons. **p < 0.01. ***p < 0.001.

### 2.3. Summary of key findings of study 1

Findings from Study 1 demonstrated that individuals’ perception of risk and effectiveness varies depending on the stage of behavioural change they find themselves in.

More precisely, the more individuals are advanced on the process of behavioural change, the closer they perceive the risks of air pollution to be, and the more effective they consider behavioural change to be. Hence, perceived risk and effectiveness are two key dimensions pushing individuals through phases, and should be effective levers to use in persuasive communication to promote behavioural change. We argue that a message focussing on the risks of air pollution in a narrative format will be effective in proximising the threat and at the same time improve efficacy appraisal, as compared to a message presenting them in a statistical format, especially for individuals in the first stages of behavioural change. To test this, we carried out Study 2.

## 3. Study 2

Based on the results of Study 1 suggesting that perceived risk and effectiveness are two levers for advancing in the stages of behavioural change, as well as on past literature on the effectiveness of narrative evidence especially for people who are less interested in an issue (e.g., Nakano and Hondo, 2023), we decided to test whether the persuasiveness of a narrative vs. statistical format varies depending on the stage of behavioural change individuals are at. Study 2 thus aims to test whether a narrative format increases pro-environmental intentions more effectively than a statistical one especially for individuals in the pre-action stage (HP1). Furthermore, Study 2 aims to understand the reason behind the persuasiveness of a narrative format, testing a mediation hypothesis arguing that the effects of a narrative vs. statistical format is mediated by its effects on individuals’ engagement with a message (HP2).

### 3.1. Method

#### 3.1.1. Participants

To estimate the sample size needed for this study, we used the average effect size published in social psychology (*d* = 0.43; Richard et al., 2003). Based on this effect size, the results obtained with G*Power indicates a sample size of at least 252 participants to achieve 80% power for an ANOVA analysis with 8 groups and 2 predictors (Format × Stage).^1^ Participants were a total of 269 adults. Data was collected via online distribution of the digitised survey (on Qualtrics). After a preliminary examination of participants’ responses, 11 participants were eliminated from further analyses. This was due to failure to correctly respond to the question “what colour is the blue sky,” leaving 258 responses for analysis.

The final sample was composed of 38% men and 62% women, aged from 14 to 69 (*M* = 30.7; *SD* = 10.49). The distribution of the participants across stages was asymmetrical, with only 11.6% of the sample in the pre-contemplation stage, 32.9% in the contemplation stage, 27.1% in the preparation stage, 8.9% in the initiation stage and 19.4% in the maintenance stage. Following the example of past research in this field (e.g., Andersson, 2020), a decision was made to combine individuals in different stages according to their position with regards to the creation of behavioural intention (in the preparation stage), which brought us to divide the sample into three groups. The pre-action group included individuals from the stages of pre-contemplation and contemplation stage (*N* = 59 exposed to a narrative message, *N* = 56 exposed to a statistical one). The preparation/intention group included individuals from the preparation stage (*N* = 33 exposed to a narrative message, *N* = 37 exposed to a statistical one). The post-action group included individuals from the initiation and maintenance stages (*N* = 35 exposed to a narrative message, *N* = 38 exposed to a statistical one).

#### 3.1.2. Procedure

Participants were recruited online, via a post published on social network groups not directly concerned with environmental protection. Data was collected between June and October 2021. After establishing informed consent, individuals were asked to indicate to which stage of behavioural change they were at with regards to pro-environmental behaviours (following the same procedure used in Study 1). Participants were randomly assigned to one of the two experimental message conditions. They were prompted to read their respective message and continue to answer a questionnaire. The measures were presented in the questionnaire the order in which they are described below (the questionnaire is reported in the section “Supplementary material”). Finally, participants were thanked and fully debriefed.

#### 3.1.3. Materials

##### 3.1.3.1. Experimental manipulation

Two experimental persuasive messages of approximately 310 words were constructed to raise awareness of the risks that air pollution presents for the environment in terms of its impact on climate change. Messages varied the type of Format (narrative vs. statistical)used to describe the evidence about the environmental risks of air pollution. The narrative evidence messages then went on to present a gender-neutral first-person account by what was designed to be a typical Western individual, “Sacha,” who described an experience of living in a city subject to a natural catastrophe in the environmental condition (e.g., “*As Sacha recounts: Shortly after the rain started the city lost power, no TV, no internet and the mobile networks weren’t working either.*”). The statistical evidence messages continued with percentages and figures of the levels of displacements and structural damages caused by the environmental consequences of air pollution (e.g., “*In 2019, 24.9 million people were displaced due to climate disasters, the highest figure recorded since 2012 and three times the number of displacements caused by conflict and violence*.”), describing the consequences of extreme climatic events (flooding) for people in general (e.g., “*The agglomeration of housing and economic activity makes people living in cities particularly vulnerable to property loss, power cuts, water contamination and sewer damage caused by flooding.*”). Both messages ended with instructions for choosing activities that can reduce one’s contribution to air pollution.

##### 3.1.3.2. Engagement

Individuals’ engagement with the message was assessed using a scale developed from previous studies’ factor analyses on narrative engagement (Busselle and Bilandzic, 2009; De Graaf et al., 2009), and was adapted for message-type stimuli. Eight items were chosen [α (*N* = 8) = 0.89], reflecting the identification and transportation types of engagement as described in the literature, measuring on a seven-point scale from *1-Strongly disagree* to *7-Strongly agree* whether they felt transported in the message (e.g., “While reading the message I found myself thinking of other things,” reversed scored) and whether they felt engaged with the individual, or the people, described in the narrative or statistical message, respectively, (e.g., “I could easily imagine myself in the situation of the [people/person] affected by air pollution described in the message”). The stimuli on identification differed slightly for participants exposed to the narrative vs. statistical message, as they asked whether they identified with “the person” described in the narrative message, or “the people” described in the statistical one.

##### 3.1.3.3. Efficacy appraisal

Efficacy Appraisal was assessed by 4 items [α (*N* = 4) = 0.91] measuring self-efficacy with two items (e.g., “I believe that I can act to reduce the air pollution in my city”), and response-efficacy with two items (e.g., “Reducing individual emissions would prevent the negative consequences of air pollution”), on a seven-point scale from *1-Strongly disagree to 7-Strongly agree*.

##### 3.1.3.4. Psychological distance of perceived risk

Psychological distance of perceived risk was measured using 8 items [α (*N* = 8) = 0.91] designed as in Study 1 to assess geographical, temporal, social distance and uncertainty in regards to the general risks of air pollution, on a seven-point scale going from *1-Strongly disagree to 7-Strongly agree*. A higher score represents greater psychological distance of the risk, lower values represent smaller distance.

##### 3.1.3.5. Behavioural intention

Behavioural intention was measured using eight items [α (*N* = 8) = 0.92] on a seven-point scale, going from *1- Not at all* to *7- Completely*, questioning participants’ intentions to engage in activities that reduce air pollution such as reducing food waste, buying local, considering different methods of personal transportation, participating in public meetings about reducing individual use of cars/motorbikes, supporting the increase of a carbon tax for individuals.

### 3.2. Results

One two-way MANOVA was carried out to analyse the influence of Format (narrative vs. statistical) and Stage (pre-action vs. preparation/intention vs. post-action) on behavioural intentions, psychological distance, engagement and efficacy appraisal. All *F* and *p*-values are reported in Table 2. Finally, a moderated mediation analysis (model 8 according to Hayes, 2012) has been carried out through the SEM programme of JASP to test whether the effect of Format on Behavioural intention, Psychological distance and Efficacy appraisal is mediated by Engagement, and whether this mediation is moderated by Stage.

**TABLE 2:** MANOVA–influence of format and stage of behavioural change (Study 2).

	*F*	*p*	η ^2^	95% CI
**Behavioural intentions**				
Format	4.06*	0.04	0.02	[−0.57; 0.59]
Stage	18.46***	0.001	0.13	[−1.99; −0.95]
Format*Stage	1.78	0.17	0.01	[−0.05; 1.42]
**Psychological distance**				
Format	6.42*	0.01	0.03	[−0.73; 0.44]
Stage	12.88***	0.001	0.09	[0.76; 1.81]
Format × Stage	1.81	0.17	0.01	[−1.41; 0.09]
**Engagement**				
Format	7.71**	0.006	0.03	[−0.37; 0.69]
Stage	1.63	0.19	0.01	[−1.18; −0.22]
Format × Stage	3.91*	0.02	0.03	[0.11; 1.46]
**Efficacy appraisal**				
Format	2.64	0.11	0.01	[−0.71; 0.43]
Stage	27.55***	0.001	0.18	[−2.35; −1.33]
Format × Stage	4.03*	0.02	0.03	[0.24; 1.68]

*p < 0.05. **p < 0.01. ***p < 0.001.

#### 3.2.1. Behavioural intentions

Format had a significant effect on participants’ intention to engage in pro-environmental actions. More precisely, individuals exposed to a narrative message reported higher intentions (*M* = 5.17; *SD* = 1.18) than individuals exposed to a statistical message (*M* = 4.81; *SD* = 1.47).

Stage of change had a significant effect on participants intentions to engage in pro-environmental actions. The Tukey’s *post-hoc* tests confirmed that individuals in stage 3 (post-action) reported higher individual intentions (*M* = 5.65, *SD* = 0.78) than individuals in stage 1 (pre-action; *M* = 4.53, *SD* = 1.67; Tukey’s *p* = 0.001) and individuals in stage 2 (preparation/intention; *M* = 5.08, SD = 0.79; Tukey’s *p* = 0.02), and that individuals in stage 2 reported higher intentions than individuals in stage 1 (Tukey’s *p* = 0.01).

The interaction Format × Stage did not have a significant effect.

#### 3.2.2. Psychological distance

Format had a significant effect on the general dimension of psychological distance: individuals in the narrative condition perceived the risks of air pollution as a closer threat (*M* = 3.07; *SD* = 1.49) than individuals in the statistical condition (*M* = 2.61; *SD* = 1.11).

Stage had a significant effect on psychological distance: individuals in the post-action stage reported the risks of air pollution as closer (*M* = 2.25; *SD* = 1.15) than individuals in the preparation/intention stage (*M* = 2.87; *SD* = 0.82; Tukey’s *p* = 0.01) and individuals in the post-action stage (*M* = 3.21; *SD* = 1.55; Tukey’s *p* < 0.001). The difference between individuals in the pre-action and preparation/intention stage was not significant (Tukey’s *p* > 0.1).

Format × Stage did not have a significant effect.

#### 3.2.3. Engagement

Format had a significant effect on engagement: individuals in the narrative condition felt more engaged (*M* = 5.18; *SD* = 1.03) than individuals in the statistical condition (*M* = 4.69; *SD* = 1.27).

Stage did not have a significant effect on engagement.

Format × Stage had a significant effect. More precisely, individuals in the pre-action stage reported higher transportation when exposed to a narrative message (*M* = 5.27; *SD* = 1.28) than when exposed to a statistical one (*M* = 4.33; *SD* = 1.65; *t*(113) = 3.43, *p* = 0.001, 95% CI = [0.39; 1.49]). This difference was not significant (all *t* < 1) for individuals in the preparation/intention stage (narrative: *M* = 5.01, *SD* = 0.72; statistical: *M* = 4.89, *SD* = 0.76) or in the post-action stage (narrative: *M* = 5.19, *SD* = 0.81; statistical: *M* = 5.03, *SD* = 0.84).

#### 3.2.4. Efficacy appraisal

Format did not have a significant effect on efficacy appraisal.

Stage significantly influenced efficacy appraisal: individuals in the pre-action stage reported lower perceived efficacy (*M* = 4.76; *SD* = 1.67) than individuals in the preparation/intention stage (*M* = 5.38; *SD* = 0.71; Tukey’s *p* = 0.003) and in the post-action stage (*M* = 6.11, *SD* = 0.75; Tukey’s *p* < 0.001). The difference between preparation/intention and post-action stages was also significant (Tukey’s *p* = 0.001).

Format × Stage also significantly affected efficacy appraisal. More precisely, individuals in the pre-action stage exposed to a narrative message reported higher perceived efficacy (*M* = 5.16; *SD* = 1.48) than those exposed to a statistical one (*M* = 4.34, *SD* = 1.77; *t*(113) = 2.71, *p* = 0.008, 95% CI = [0.22; 1.42]). This difference was not significant (all *t* < 1) for individuals in the preparation/intention stage (narrative: *M* = 5.42, *SD* = 0.64; statistical: *M* = 5.34, *SD* = 0.78) or in the post-action stage (narrative: *M* = 6.04, *SD* = 0.75; statistical: *M* = 6.18, *SD* = 0.75).

#### 3.2.5. Moderated mediation

**FIGURE 1:**
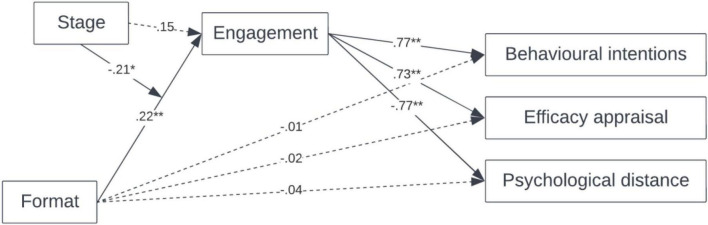
Standardised structural relations; moderated mediation model (Study 2). *p < 0.05. **p < 0.01.

We conducted structural equation modelling using JASP; we tested model 8 (see Igartua and Hayes, 2021), with Format as the independent variable, Stage as the moderating variable, Engagement as the mediating variable, and Behavioural intention, Efficacy appraisal and Psychological distance as dependent variables. The model is presented in Figure 1.

The direct effect of Format on Engagement is significant (β = 0.22, SE = 0.07, *p* = 0.002, 95% CI [0.078, 0.361]), as the interactive effect of Format × Stage (β = −0.21, SE = 0.08, *p* = 0.02, 95% CI [−0.368, −0.038]), while the direct effect of Stage on Engagement is not significant (β = 0.15, SE = 0.08, *p* = 0.08, 95% CI [−0.02, 0.311]). The direct effect of Engagement on Behavioural intentions is significant (β = 0.77, SE = 0.05, *p* < 0.001, 95% CI [0.667, 0.875]), as well as its direct effect on Efficacy appraisal (β = 0.73, SE = 0.06, *p* < 0.001, 95% CI [0.613, 0.837]) and on Psychological distance (β = −0.77, SE = 0.05, *p* < 0.001, 95% CI [−0.873, −0.669]). The indirect effect of Format through Engagement on Behavioural Intentions is not significant (β = −0.01, SE = 0.06, *p* = 0.87, 95% CI [−0.133, 0.113]), as the indirect effect of Format through Engagement on Efficacy Appraisal (β = −0.02, SE = 0.07, *p* = 0.83, 95% CI [−0.147, 0.117]) and on Psychological distance (β = −0.04, SE = 0.06, *p* = 0.51, 95% CI [−0.160, 0.080]).

### 3.3. Summary of key findings of study 2

Findings from Study 2 demonstrate that individuals have different risk and effectiveness perceptions depending on the stage of behavioural change that they are at, with people already engaged in behavioural change being more sensitive to environmental issues and more motivated to engage in further environmental efforts.

The data also suggests that while the influence of the format of evidence on behavioural intentions does not vary depending on the stage of change of individuals, only individuals who are not yet engaged in changing their behaviours in favour of the environment are highly sensitive to the effect that format has on their efficacy appraisal. The theoretical and practical implications of these findings are further discussed in the general discussion.

## 4. General discussion

Findings from the research presented in this paper demonstrate that individuals in different stages of the process of behavioural change have different risk and efficacy appraisals when it comes to air pollution, and are thus affected differently by narrative persuasion. Indeed, data from Study 1 confirms that the more individuals are advanced on the process of behavioural change, and are engaging or have already engaged in pro-environmental behaviours, the closer they perceive the environmental risks of air pollution to be, and the more effective they consider pro-environmental actions to be in improving air quality. This is in line with past research and models in the field of behavioural change explaining how individuals’ decision to engage in specific new behaviours when facing a threat directly depends on individuals’ appraisal of the threat but also of the effectiveness of alternative behaviour to deal with such a threat (Witte and Allen, 2000; Kothe et al., 2019; Shafiei and Maleksaeidi, 2020). Indeed, according to these models, individuals who do not perceive a threat as close and severe, and/or do not consider the alternative behaviour as effective to deal with the threat, do not engage in behavioural change. By confirming that individuals who are engaging or have engaged in behavioural change perceive higher threat and higher efficacy than individuals who have not yet engaged in behavioural change, our data confirms that these two dimensions are fundamental to push people to advance through the different stages of behavioural change.

Data from Study 1 confirm that perceived risk and perceived efficacy are two key dimensions differentiating the different phases of behavioural change, suggesting that they could be used as levers to push individuals to advance through phases, via narrative persuasion. The findings of Study 2 demonstrated in fact that the stage of behavioural change determines the sensibility of citizens to the influence of the evidence format used in a communication about the environmental risks of air pollution. Indeed, individuals in a phase of pre-contemplation and contemplation reported closer psychological distance and higher efficacy appraisal when receiving a narrative communication rather than a statistical one, but this difference was not significant for individuals in the action and post-action stages. This is in line with past research demonstrating that individuals in these stages are ambivalent about their current behaviour, which makes them more sensitive to external influences (Forward, 2014; Andersson, 2020). This could also explain why past research has found heterogeneous results with regards to the effectiveness of narrative vs. factual communications in promoting pro-environmental behaviour, with some studies finding no significant difference with regards to transportation and behavioural intention (e.g., Jones, 2014).

A question remains concerning the reason why statistical format seems to not be effective in any of the stages of behavioural change. Indeed, presenting scientific evidence is important to explain how certain conclusions have been reached through data, although past empirical research has demonstrated that the ability to understand statistics and numbers varies greatly among individuals (Reyna et al., 2009; Peters, 2012; Hopp, 2015), which could explain the lack of persuasiveness when exposing a general sample to a statistical format of evidence. The research presented in this paper does not allow us to draw a conclusion on the non-persuasiveness of the statistical format, because individuals’ numeracy and/or mathematical expertise was not measured, nor was the understanding of the statistics presented in general. Further research should thus focus not only on the reasons why narrative format is persuasive, but also on the reasons why statistical format is not, and whether this depend on the expertise and knowledge of the population exposed to the messages.

Our findings about the effectiveness of narrative persuasion are however less explicit when considering behavioural intentions, as no interactive effect is obtained. Given the limited overall sample of Study 2 (especially considering the sub-samples in each stage of behavioural change), modelling analyses could not be carried out (Jackson, 2003); future research on this topic should aim at collecting data from a larger sample in order to allow for structural equation modelling guided by hypotheses well-grounded in theory and past research. A narrative message seems more effective than a statistical one boosting participants’ intention to engage in pro-environmental behaviours, regardless of the stage of behavioural change individuals are in. This could be due to the «habitual» nature of the targetted behaviour, driving. indeed, it has been argued that one of the conditions to modify habits is that individuals perceive the positive benefits of behavioural change in a short-term perspective (Jager, 2003), and that procrastination of behavioural change is reduced when individuals can imagine concretely the new behaviour or task (McCrea et al., 2008). It is thus possible to suppose that a narrative format would improve the ability of individuals to imagine short term benefits in a concrete way, thus influencing behavioural intentions for all individuals regardless of the stage of behavioural change they are in. Future research should explore further the reasons why a narrative message is more effective even for people who are in the process of changing their behaviour or have changed it already.

One further limitation of Study 2 concerns the influence of format on individuals’ engagement with a message. This effect could have been biassed by the way the two formats (statistical and narrative) have been phrased in the messages, specifically when considering narrative identification. Indeed, if the narrative message presented a specific character and his experience with flooding, mentioning the character six times and thus giving several opportunities to participants to identify with the character, the statistical message only mentioned “people” and their experience a couple of times. This incongruence could have resulted in further identification with the narrative than with the statistical format. The two messages were not pre-tested, and this is one of the main methodological limitations of Study 2. Future studies need to further analyse whether a statistical message offering the same opportunities to identify with the people described as a narrative message also results in higher narrative engagement.

It is important to consider that the generalisation of our data could be limited by the fact that the participants in Study 1 and Study 2 were not equally distributed across the five stages identified by the TTM (Prochaska and DiClemente, 1984; Prochaska et al., 2015). The difficulty of finding individuals in the pre-contemplation stage, which also features in past studies (e.g., Forward, 2014; Andersson, 2020) might depend on a general increase of environmental awareness that developed in the last decades (Special Eurobarometer 468, 2017; Bandura and Cherry, 2020). This difficulty brings us to question the pertinence of dividing the process of behavioural change in favour of the environment in five stages based on “arbitrary time periods” (Sutton, 2000), which has been previously criticised (Weinstein et al., 1998; Sutton, 2000; Littell and Girvin, 2002; Wilson and Schlam, 2004). Further research could rather focus on the original three phases of behavioural change as described by Lewin (1951): unfreezing, changing and refreezing. Further research should thus explore whether the increase of environmental awareness in Western societies calls for the development of a new model of the stages of behavioural change to be adopted in experimental work as well as in interventions to promote pro-environmental behaviours.

Overall, the present research addresses two main gaps in the literature with regards, on one hand, to the understanding of how risk- and efficacy-perceptions vary across stages of behavioural change, and on the other hand to the study of the persuasiveness of narrative evidence. Indeed, findings from study 1 demonstrate that individuals in the first stages of behavioural change perceive the environmental consequences of air pollution to be psychologically distant (which triggers low perception of risk and vulnerability) and the behaviours to improve air quality to be less effective than individuals in the more advanced stages of behavioural change. This in line with past research in the field of health promotion (e.g., Marshall and Biddle, 2001), confirming that also in the field of pro-environmental behaviour, perceived risk and perceived effectiveness are two levers that could push individual to advance through stages of behavioural change. Furthermore, findings from study 2 advance the research on narrative persuasion in the field of pro-environmental behavioural change, suggesting that the effectiveness of narrative vs. statistical formats of risk information depends on the way individuals are positioned with regards to the process of engaging in pro-environmental behavioural change. This offers practical advice for the development of education and communication programmes, contributing to research demonstrating that different communication strategies might be more or less effective in promoting environmental awareness and behavioural change depending on the segment of the targetted population.

## Data availability statement

The raw data supporting the conclusions of this article will be made available by the authors, without undue reservation.

## Author contributions

LB was in charge of the concept and design of the studies, data analysis, and writing of the article. MC has made a substantial contribution for the development of the methods and as well as data collection and analysis. FM revised the article critically for important intellectual content as well as data analysis and interpretation. All authors contributed to the article and approved the submitted version.

## Footnotes

1 In order to correspond to Study 1, we initially decided to consider participants as distributed over four–and not five–stages of behavioural change. Finally, they were categorised into three distinct change groups (see explanations below).
