# Supplementary material for: When narratives speak louder than numbers: the effects of narrative persuasion across the stages of behavioural change to reduce air pollution
Source: Front Psychol. 2023 Apr 26;14:1072187. doi: 10.3389/fpsyg.2023.1072187 (PMC10171234; doi:10.3389/fpsyg.2023.1072187)
Supplement: Supplementary file 1 [file Table_1.DOC]

**Supplementary Material**

**“When narratives speak louder than numbers: the effects of narrative persuasion across the stages of behavioural change to reduce air pollution”**

**Measurement of psychological distance (Study 1 and 2)**

Please rate your agreement with the following items, on a scale from 1 – Strongly disagree, to 7 – Strongly agree.
*(The presentation of the items was randomised in both studies)*

1. The environmental consequences of air pollution are more likely to impact faraway countries
2. My local area is largely immune to the environmental consequences of air pollution
3. The environmental consequences of air pollution are an immediate threat affecting people right now
4. The recent impacts of air pollution on the environment mean we must tackle the issue now
5. The negative consequences of air pollution for the environment will definitely affect me and my family
6. I don’t think that the environmental issues caused by air pollution will significantly impact people I know
7. The environmental consequences of air pollution are widely exaggerated
8. I am certain that air pollution has negative environmental consequences

**Measurement of behavioural effectiveness (Study 1)**

Please rate the degree to which you perceive the following behaviours to be effective in improving air quality, on a scale from 1 – Not at all effective to 7 – Completely effective.
*(The presentation of the items was randomised)*

1. Reduce the use of individual cars
2. Recycling
3. Buying local products
4. Buying seasonal products
5. Financial incentives for individuals to reduce their emissions
6. Financial incentives for industries to reduce their emissions
7. Implementing a carbon tax for highly polluting industries
8. Reduce the access of motorised vehicles to big cities
9. Creating a low emission zone in cities
10. Forbidding intra-national flights
11. Convincing people around you to reduce their emissions
12. Supporting collaborative projects to reintroduce nature in cities

**Measurement of behavioural change stage (Study 1 and 2)**

Which statement best describes how you travel in everyday life?
*(The presentation of the different sentences to choose one from was randomised in both studies)*

- I use the car for the most part and do not intend to change the mode of transport within the next six months;
- I am using the car for the most part, but I am considering replacing some car journeys with other modes within the next six months;
- I am using the car for the most part, but have begun trying other modes instead the last six months;
- For the past six months, I have only used the car as a complement to other means of transport;
- For the past six months, I have only used modes other than cars.

Measurement of engagement (Study 2)

| *(To participants exposed to a narrative format)* | *(To participants exposed to a statistical format)* |
| --- | --- |
| The following statements are about your experience of reading the previous text and your thoughts about the person affected by the information that was presented. | The following statements are about your experience of reading the previous text and your thoughts about the people affected by the information that was presented. |
| Please rate your agreement with each statement on a scale from 1 – Strongly disagree to 7 – Strongly agree. | |
| 1. At times I had a hard time making sense of what the message was telling me | |
| 1. I found my mind wandering while reading the message | |
| 1. While reading the message, I found myself thinking of other things | |
| 1. My understanding of the person affected by air pollution is unclear | 4. My understanding of the people affected by air pollution is unclear |
| 1. The message affected me emotionally | |
| 1. I felt sorry for the person affected by air pollution referred to in the message | 6. I felt sorry for the people affected by air pollution referred to in the message |
| 1. While reading the message, I had a feeling of what the person affected by air pollution go through | 7. While reading the message, I had a feeling of what the people affected by air pollution go through |
| 1. I could easily imagine myself in the situation of the person affected by air pollution described in the message | 8. I could easily imagine myself in the situation of the people affected by air pollution described in the message |

**Measurement of efficacy appraisal (Study 2)**

Please rate how you feel about the following statements regarding actions to reduce air pollution, on a scale from 1 – Strongli disagree to 7 – Strongly agree.

1. I believe that I can act to reduce the air pollution in my city
2. I feel able to rise to the challenge of reducing the greenhouse gases that my activity is responsible for
3. Reducing the emissions that individuals are responsible for would improve air quality
4. Reducing individual emissions would prevent air pollution

**Measurement of behavioural intentions (Study 2)**

Please rate your intention to undertake the following actions in the future, on a scale from 1 – Not at all, to 7 - Completely.

1. I intend to increase my use of eco-responsible modes of transportation (walking, cycling, public transport)
2. I intend to buy products from local providers
3. I intend to increase the accuracy of my recycling
4. I intend to reduce my food waste or dispose of it in an environmentally responsible manner
5. I intend to sign petitions in support of stricter industry regulations regarding food waste
6. I intend to volunteer my time to projects that help reintroduce nature into cities
7. I intend to participate in public meetings about reducing individual use of cars/motorbikes
8. I intend to support the increase of a carbon tax for individuals

**Experimental manipulation (Study 2)**

The messages presented to individuals started and ended with the same sections, however in the middle section they either presented a personal account of an individual experiencing the environmental consequences of air pollution (narrative format) or giving numbers and percentages concerning the structural damages caused by the environmental consequences of air pollution (statistical format).

| **Burning, Flooding, Changing Lives: What Can We Do?**  Carbon dioxide (CO2) occurs naturally in the atmosphere but has increased since the Industrial Revolution. As CO2 levels have risen, so have its effects on air pollution. Although the categorisation of CO2 as an air pollutant is debated, air pollution patterns are changing in several urbanised areas of the world due to climate change, primarily caused by the increase of CO2 emissions. The primary causes are deforestation and fossil fuel burning, used to propagate activities such as transportation and household energy use. Increased CO2 emissions have led to the greenhouse effect in which the Earth’s global temperature is offset. An increase by 2 degrees may seem small but it triggers a significant increase in accumulated heat. This extra heat drives temperature extremes, intensifying heavy rainfall, rising sea levels and heat waves leading to increased occurrences of wildfires, floods and drought. | |
| --- | --- |
| ***Narrative format*** | ***Statistical format*** |
| Personal accounts testify to the effects that more people may start to experience should CO2 levels rise without action. As Sacha recounts: “Shortly after the rain started the city lost power, no TV, no internet and the mobile networks weren’t working either. The intensity of the rainfall dawned on me when I heard water flowing outside my door… I live on the fourth floor. It was unlikely that the water had risen so quickly, looking out to the corridor I saw it gushing down the stairs. The building’s terrace had filled with water so suddenly that the drains couldn’t cope. On the ground, cars were almost completely submerged. No electricity meant no tap water since the pumps weren’t functioning. I had to wade waist-deep to get bottled water.” | In 2019, 24.9 million people were displaced due to climate disasters, the highest figure recorded since 2012 and three times the number of displacements caused by conflict and violence. Over 100 developed cities are vulnerable to impacts by floods and heatwaves. The increase in heatwave duration ranges from 4% to 69%. Some cities are predicted to experience drought conditions that are up to 14 times worse than historically recorded. Developed island countries and river cities have flood projections of more than a 50% increase of their 10 year high river flow. The agglomeration of housing and economic activity makes people living in cities particularly vulnerable to property loss, power cuts, water contamination and sewer damage caused by flooding. |
| There are simple lifestyle changes that you can do to make a difference. Choosing to walk, cycle or take public/shared transportation can reduce your carbon footprint. Ensure that your home is energy efficient and turn off lights and appliances when not in use. Buy fewer items new, consider instead used or recycled products. Protect your environment: be a part of the solution to air pollution. | |
